# Therapeutic options for chronic myeloid leukemia following the failure of second-generation tyrosine kinase inhibitor therapy

**DOI:** 10.3389/fonc.2024.1446517

**Published:** 2024-07-29

**Authors:** Binsah George, Kok Hoe Chan, Adan Rios

**Affiliations:** Division of Hematology/Oncology, Department of Internal Medicine, The University of Texas Health Science Center at Houston, Houston, TX, United States

**Keywords:** chronic myeloid leukemia, second-generation tyrosine kinase inhibitor, CML, TKI, failure, therapeutic options

## Abstract

The management of chronic myeloid leukemia in the chronic phase (CML-CP) has witnessed significant advancements since the identification of a common chromosomal translocation anomaly involving chromosomes 9 and 22, which results in the formation of the Philadelphia chromosome driven by the BCR-ABL1 fusion protein. This discovery paved the way for the development of tyrosine kinase inhibitors (TKIs) that target the adenosine triphosphate (ATP) binding site of ABL1 through the BCR-ABL-1 fusion protein. Following the approval of Imatinib by the Food and Drug Administration (FDA) as the first TKI for CML treatment in 2001, the median overall survival (OS) for chronic phase CML (CML-CP) has significantly improved, approaching that of the general population. However, achieving this milestone crucially depends on reaching certain treatment response milestones. Since the introduction of imatinib, five additional TKIs have been approved for CML-CP treatment. Despite the availability of these treatments, many patients may experience treatment failure and require multiple lines of therapy due to factors such as the emergence of resistance, such as mutations in the ATP binding site of ABL, or intolerance to therapy. This review will primarily focus on exploring treatment options for patients who fail second-generation TKI therapy due to true resistance.

## Introduction

The primary objective of treating chronic phase chronic myeloid leukemia (CML-CP) is to prevent progression to more aggressive accelerated or blast phase CML, regardless of the tyrosine kinase inhibitor (TKI) line, enabling patients to achieve a life expectancy similar to the general population (1). Since the approval of the first TKI, imatinib, in 2000, the 10-year overall survival (OS) trajectory for CML-CP has increased from 20% to 80% (2). Presently, six TKIs are approved for CP-CML treatment: first-generation TKI imatinib; second-generation (2G) TKIs nilotinib, dasatinib, and bosutinib; and third-generation (3G) TKIs ponatinib and asciminib. For the majority of CP-CML patients, imatinib is recommended as the first-line (1L) therapy for long-term disease control (3). Imatinib is generally associated with fewer cardiovascular and arterio-occlusive events compared to 2G TKIs (4–6). However, due to various reasons, including disease-related factors or the pursuit of a higher and faster treatment-free remission (TFR), some patients may opt for a second-generation TKI as 1L treatment. Nevertheless, there is currently no evidence indicating a survival advantage of second-generation TKIs over imatinib (7–9).

In major multicenter, phase 3 clinical trials comparing imatinib to 2G TKIs in newly diagnosed CML-CP, such as dasatinib (DASISION), nilotinib (ENESTnd), and bosutinib (BFORE), higher rates of complete cytogenetic response (CCyR) and major molecular response (MMR) were observed with 2G TKIs in the 1L setting (4, 5, 10). Dasatinib demonstrated CCyR and MMR rates at 12 months of 77% and 46%, respectively, versus 66% (P=0.007) and 28% (P=0.0001) with imatinib. Nilotinib 400 mg showed a confirmed MMR rate at 12 months of 43% versus 22% (P=0.0001) with imatinib. Bosutinib exhibited CCyR and MMR rates at 12 months of 77% and 47%, respectively, versus 66% (P=0.0075) and 37% (P=0.0200) with imatinib. Cumulative 5-year MR4.5 rates were as follows: dasatinib 42% versus imatinib 33% (P=0.0251); nilotinib 52% versus imatinib 31% (P=0.0001); bosutinib 47.4% versus 36.6% (4–6).

Treatment failure may result from either primary resistance, defined as the inability to achieve target molecular responses within the specified duration, or secondary resistance, characterized by the loss of prior response. Intolerance is defined as recurrent grade ≥ 3 hematological toxicity or ≥ 2 nonhematological toxicity requiring discontinuation despite dose reduction (4). Discontinuation rates due to adverse events (AEs) were reported as follows: IRIS (7%), DASISION (16%), ENESTnd (12%) (nilotinib 300mg twice daily), BFORE (25%), PACE (21%), and ASCEMBL (5.8%) (4–6, 11, 12). Each TKI has unique toxicity profiles, so exercising caution when selecting an appropriate TKI can improve compliance and mitigate side effects.

After genuine resistance to 2G TKIs, a more potent therapy is needed. The choice should be based on disease-specific factors such as mutational profile, cytogenetics, risk profile, and adverse events of specific and prior TKI therapy. Current recommendations include switching to another 2G TKI or a 3G TKI, with plans for early allogeneic stem cell transplantation or enrollment in a clinical trial if treatment milestones or deep molecular responses (DMRs) are not achieved or maintained (13–16). However, data on precise clinical guidance post-2G TKI failure, whether used as 1L or second line (2L), are limited. This review will provide insights into clinical evidence and guidance, including new therapeutics in clinical trials, following 2G TKI failure due to genuine resistance.

## Mechanisms of resistance to second generation TKI

Resistance to therapy most commonly arises from either novel mutations in the BCR::ABL1 gene, such as mutations in the kinase domain or overexpression/amplification of BCR::ABL1, disrupting TKI binding. Mutations account for resistance in approximately one-third of resistant CP patients. Resistance can also occur via non-BCR::ABL1 mechanisms, including SRC kinases or increased P-glycoprotein efflux pump activity, clonal evolution, reduced levels of human organic cation transporter (hoct1) leading to decreased TKI influx, or increased levels of the multi-drug exporter of the ATP binding cassette (17–21). Genetic aberrations in ASXL1 were found to be significantly higher in TKI-resistant patients treated with imatinib, raising concerns about possible preexisting ASXL1 mutations in the BCR::ABL1-positive leukemic clone impacting the clinical response to imatinib. However, further studies are needed to validate this correlation due to the limited sample size (22).

Mutations at diagnosis are rare but can emerge in patients due to noncompliance and may develop resistance to TKI therapy (23–27), or after multiple sequential TKI therapies, associated with decreased response and worse overall survival (18, 28). Mutations usually involve acquired point mutations in the BCR::ABL kinase domain (18, 28). Whole-genome sequencing with the identification of mutated genes such as ASXL1 and TP53 in CP-CML may hold prognostic and predictive significance, requiring further investigation in clinical management (29).

Sequential therapies with TKIs increase the vulnerability to the emergence of compound mutations, with two paired mutations occurring in 76% of cases, and triple (10.6%) and quadruple (1.5%) mutations within the same BCR::ABL1 allele. Unfortunately, these are usually insensitive even to third-generation TKIs (30–32). Ponatinib, a high-affinity pan BCR-ABL1 inhibitor, can suppress all single mutants in the BCR::ABL1 domain, including T315I. However, the emergence of compound mutations in a BCR::ABL1 allele, especially those involving T315I (e.g., Y253H/T315I, E2455V/T315I), may confer ponatinib resistance, even at a high dose of 45mg once a day (31, 33, 34). A clinical consideration is the relevant combination of asciminib and ponatinib, which appears effective in overcoming compound mutations involving T315I and reducing ponatinib toxicity (34).

## Criteria for therapy failure

Failure can be categorized as either true resistance or intolerance. However, the focus of this paper will be on true resistance to 2G TKIs. During therapy for CML-CP, there are specific recommendations regarding achieving target molecular responses at different time points (3, 6, 12 months) by measuring BCR::ABL transcript levels using real-time reverse transcriptase polymerase chain reaction (RT-PCR), as outlined in international standards (IS) (35, 36).

The 2013 European Leukemia Net (ELN) definition had different criteria for failure to first- and second-line TKIs, with less stringent instructions after failing second-line therapy. However, the 2020 ELN definition considers the presence of a mutation and failure to achieve a BCR::ABL1IS ≤1% or CCyR at 12 months as treatment failure, encompassing those receiving second-line TKIs. The ELN 2020 criteria are summarized in **Table 1** (37).

**Table 1:** ELN 2020 definition of failure to 1L and 2L treatment (37).

Time	Definition of TKI Failure
3 months	BCR::ABL1 (IS) > 10% if confirmed within 1–3 months
6 months	BCR::ABL1 (IS) > 10%
12 months	BCR::ABL1 (IS) > 1%
Any time	BCR::ABL1 (IS) > 1%, resistance mutations, high risk ACA

IL, First line; 2L, Second line; IS, international standard; ACA, additional chromosomal abnormalities; TKI, Tyrosine kinase inhibitor.

As generally acknowledged, second-generation TKIs achieve faster rates of CCyR at early time points compared to imatinib. Therefore, applying ELN 2020 criteria to the use of second-generation TKIs as initial therapy in CML-CP may not be optimal. Previously, studies by Jabbour et al. and more recently by Sasaki et al. have suggested that patients on 2G TKIs as frontline therapy had worse survival outcomes if an earlier switch to ponatinib or a novel TKI was not initiated when a 3-month BCR::ABL ≤10% and 6–12 month BCR::ABL1IS ≤1% were not achieved (38, 39). These guidelines establish treatment change targets to mitigate the risk of disease progression.

Treatment failure may result from either primary resistance, defined as the inability to achieve target molecular responses within the allocated duration (**Table 1**), or secondary resistance, characterized by the loss of prior response. The loss of CHR or CCyR necessitates a therapy switch, but the loss of MMR within the context of sustained CCyR allows for less precise interpretation (21, 40).

## Therapeutic options after resistance to first-line second-generation TKI and outcomes

While imatinib is considered the safest option (41), it does not effectively inhibit several BCR::ABL mutations (42), with the exception of the gatekeeper mutation T315I, which is sensitive to ponatinib and asciminib (19, 21). By five years, 30–55% of patients treated with 2G TKIs achieve a 4.5 log reduction (MR4.5, BCR::ABL1IS <0.0032% IS), compared to approximately 30% treated with imatinib (4, 5). Although 2G and 3G TKIs have advantages over imatinib in achieving a faster and deeper response, there is currently no data confirming higher rates of cure (15, 37, 42).

Approximately 50% of patients with CML-CP treated with imatinib will switch therapy within five years, compared to 30–40% when treated with frontline 2G TKI. Among these, nearly 15%-25% change due to true resistance to imatinib associated with the T315I mutation, while only 5–7% are due to intolerance (4, 5, 43). At five years, the rates of resistance for nilotinib (ENESTend), dasatinib (DASISION), and bosutinib (BFORE) as first-line therapies are 23%, 26%, and 5.6%, respectively (4, 5, 44–46). The outcomes of 2G TKI as first-line therapy are outlined in **Table 2**.

**Table 2:** Outcomes of 2G TKI post imatinib failure.

TKI	CHR (%)	CCyR (%)	MMR (%)	PFS (%)	OS (%)	Follow up(months)
Dasatinib (47)	89	44	40–43‡	45–56	76	72
Nilotinib (48)	77	45	NA	57	78	≥48
Bosutinib (49)	86	48	46	NA	73	≥108

CHR, complete hematological response; CCyR, complete cytogenetic response; MMR, major molecular response; OS, overall survival; ‡ Different dosing of dasatinib, 70–140 mg daily. NA, not available.

A minority of patients are resistant to second-generation TKIs in the first-line setting and represent a population with a poor prognosis requiring a switch to alternative therapy. Failing a 2G TKI in the first-line setting is adverse compared to failing it in the second-line setting (21). Providers often choose 2G TKIs as first-line therapy as they provide higher rates of complete CHR, CCyR, and MMR and are more tolerable than high-dose imatinib (50–52).

Although there are no prospective trials and the patient numbers are low, the rates of CCyR after failure of imatinib and dasatinib were 27% and 20%, respectively, with nilotinib and bosutinib when used as third-line therapy, but were higher with sequential ponatinib at 54%. When patients failed imatinib and nilotinib, the CCyR rates were 25–26% with third-line therapy with dasatinib or bosutinib, whereas they were 67% when switched to ponatinib. Similar results were observed in patients who failed a 2G TKI and switched to an alternative 2G TKI, resulting in CCyR rates of 22–26%, compared to 60% with ponatinib, including T315I and non-T315I mutated patients (53, 54). Hence, an alternative 2G TKI has limited value after resistance to another 2G TKI in the absence of mutations, and few patients remained on treatment, indicating considerable failure across studies (55–60).

In a retrospective study of 62 patients with a median follow-up of 14 months, treated with fourth-line bosutinib post-failure to first-generation and remaining 2G TKIs, the probability of achieving and maintaining a CCyR and MMR was 25% and 24%, respectively. This number further decreased to 14% to achieve an MMR if patients were not in a CCyR at the time of starting bosutinib (61).

Recently, Kantarjian et al. demonstrated sustained high response and survival outcomes with ponatinib in patients resistant to 2G TKIs, regardless of T315I status, enrolled in the PACE (Ponatinib Philadelphia chromosome-positive acute lymphoblastic leukemia and CML Evaluation) and OPTIC (Optimizing Ponatinib Treatment in CP-CML) studies. The progression-free survival (PFS) and overall survival (OS) were 68% and 85% in the PACE study, and 80% and 91% in the OPTIC study (62).

Therefore, in the case of resistance to a 2G TKI due to a specific mutation, other 2G TKIs could be considered. For instance, following resistance to dasatinib, nilotinib, or bosutinib could be options depending on specific mutations, patient comorbidities, compliance, drug-drug interactions, and prior adverse effects. However, in accordance with ELN 2020 guidelines, an earlier use of ponatinib should be considered in all eligible patients without significant cardiovascular disease, as they are twice as likely to achieve a CCyR when treated with ponatinib than with another 2G TKI (62–65).

Multiple factors need to be assessed for resistance to a 2G TKI, but mutational analysis should be performed primarily, and discussions regarding finding a suitable donor should be initiated. Next-generation sequencing (NGS) is a more sensitive technique than Sanger sequencing and can detect low-level mutations and compound mutations. However, resistance to TKIs may not solely be due to low-level mutations and does not guide TKI selection unless it involves T315I, which necessitates ponatinib or a higher dose of asciminib (66). Detecting compound mutations, especially Y253H/T315I and E2455V/T315I, should prompt a search for a donor for allogeneic stem cell transplantation (30, 67, 68).

Cross tolerance is uncommon, and side effects usually change upon switching therapy, except for myelosuppression, which can persist across TKIs (69–71). However, patients who demonstrate failure to multiple TKIs and switch to an alternative 2G TKI may not experience high response rates, and if achieved, the response is not usually durable (53–55). Achieving a CCyR at three months is independently associated with event-free survival (EFS) and OS; hence, in patients who are not candidates for transplantation, maintaining a CCyR with different TKIs could be a therapeutic goal rather than aiming for MMR or a deeper response (72). However, following resistance to a first- or second-generation TKI, a reduced CCyR is observed.

## Second line TKI: efficacy and outcomes

The debate over the best strategy for initial therapy ranges from starting with a 2G TKI for a quicker and more profound response to switching to a 2G TKI after an inadequate response to imatinib. MMR is generally regarded as a surrogate for survival, and using 2G TKIs as initial therapy has not demonstrated improvements in overall survival (OS), progression-free survival (PFS), or treatment-free remission (TFR) (7, 62, 73–75).

While imatinib is commonly used worldwide as the first-line TKI, an increasing number of physicians are choosing 2G TKIs as first-line therapy to achieve a faster and deeper remission, with the aim of achieving TFR. However, TFR is only considered appropriate if patients achieve a MMR with sustained deep remission, typically defined as a 4-log decrease in BCR::ABL transcript levels from a standardized baseline, corresponding to a PCR <0.01% on the international scale (IS) (76, 77).

In a cohort of 113 patients, fewer than 10% achieved a CCyR at 3–6 months and eventually attained a major cytogenetic response (MCyR) at 12 months after receiving a 2G TKI (dasatinib/nilotinib) (78).

In patients with imatinib failure, the T315I mutation was reported in 10–27%, however, in the second-line setting, it was observed in 9–53% (43). Currently, FDA-approved treatment options for the T315I mutation include ponatinib, asciminib, omacetaxine (only approved in the USA), and allo-SCT (19, 33, 79). Olverembatinib (HQP1351) is in clinical trials and has shown activity against T315I.

Treatment with 2G TKIs after imatinib failure can result in high response rates and is a more effective option compared to higher doses of imatinib (800mg daily) in achieving higher CCyR and MMR (50, 51, 80, 81). **Table 2** illustrates outcomes with second-line TKIs after imatinib resistance.

## PACE and OPTIC study and real-world ponatinib data

Ponatinib, a 3G TKI, is approved for patients with the T315I mutation or those resistant or intolerant to at least two TKIs in CML-CP (82, 83). In the 5-year follow-up of the pivotal PACE trial (Ponatinib Philadelphia chromosome-positive acute lymphoblastic leukemia and CML Evaluation), where a heavily pretreated cohort of patients resistant or intolerant to dasatinib or nilotinib, or with the T315I mutation, was enrolled, significant findings were observed (84, 85). Out of 267 evaluable patients with CML-CP and after a median follow-up of 56.8 months and median duration of treatment of 32.1 months, 144 (54%) achieved a CCyR, 108 (40%) achieved an MMR, and 64 (24%) achieved MR4.5. Of those who achieved an MCyR at 12 months and an MMR at any time, 82% and 59% of patients, respectively, maintained responses at 5 years. The median times to MCyR, CCyR, and MMR among those who achieved the response were 2.8, 2.9, and 5.5 months, respectively. The Kaplan-Meier estimated PFS and OS at 5 years were 53% and 73%, respectively (82).

To better determine the optimal dose of ponatinib while balancing potency and safety, the phase 2 open-label OPTIC study (Optimizing Ponatinib Treatment in CP-CML) was conducted (NCT02467270), where patients were randomized to receive either ponatinib at 45 mg daily (cohort A), 30 mg daily (cohort B), or 15 mg daily (cohort C). Preliminary analyses showed varying rates of achieving BCR::ABL1IS ≤ 1% (MMR) at 12 months across the cohorts (84, 85). At the recent 3-year follow-up update, MMR at 36 months was observed in different percentages across the cohorts (86). Adverse events occurred in varying percentages across the cohorts, with grade ≥3 adverse events reported in a smaller subset. Discontinuations due to treatment-emergent adverse events occurred in differing percentages across the cohorts, with a minimal number of deaths reported (12, 84, 86).

Recent data from the Belgian registry on 33 CML-CP patients previously treated with at least two TKIs showed promising results with ponatinib, albeit with some incidence of therapy discontinuation due to side effects (87). Similarly, real-life experience from Italy on treating patients with ponatinib demonstrated favorable responses but also highlighted therapy discontinuation rates due to resistance or intolerance (88).

In the US registry for CML-CP patients receiving ponatinib, various starting doses were observed, with preferences and recommendations outlined by ELN 2020 guidelines based on cardiovascular risk factors and resistance profiles (37, 89–91). The use of aspirin as primary thromboprophylaxis while on TKI remains uncertain (92, 93).

## Asciminib: a first-in-class allosteric inhibitor

Asciminib, a pioneering selective allosteric BCR-ABL1 inhibitor, represents a distinct mechanism of action compared to currently available TKIs. FDA approval in October 2021 for third-line use or in patients harboring the T315I mutation underscores its significance (82, 83). By mimicking the myristoyl peptide, asciminib precisely targets the ABL Myristoyl Pocket (STAMP inhibitor), thereby restoring the inactive form of the kinase during the 9;22 translocation without affecting the ATP binding site. This unique mechanism grants asciminib activity against various ATP site resistance mutations, including the gatekeeper T315I mutation, catalytic site, and P-loop mutation (excluding F359) (19, 94, 95).

Asciminib’s efficacy was initially explored in the phase 1 CABL001X2101 trial, where it was assessed as monotherapy or in combination with nilotinib or dasatinib in CML-CP or CML-AP as third-line therapy or in the second line for T315I mutation. Results from the monotherapy cohort of 150 patients demonstrated promising outcomes, with significant proportions achieving MMRs at various time points (19). Subsequent updated results from 115 patients, after nearly four years of follow-up, revealed continued efficacy, with a considerable proportion maintaining MMRs and MR4s (96).

Common grade ≥3 adverse events included increased pancreatic enzymes, thrombocytopenia, hypertension, and neutropenia, while musculoskeletal pain, upper respiratory tract infection, and fatigue were frequent all-grade adverse events (96).

Furthermore, an expanded cohort of the phase 1 study evaluated asciminib in 52 T315I-positive CML-CP patients at an escalated dose of 200mg BID, showing notable MMR rates and a manageable safety profile (19, 97).

In a subgroup analysis of heavily pretreated patients, asciminib monotherapy demonstrated effectiveness in achieving MMRs, MR4s, and MR4.5s, with a favorable safety profile (96, 98).

These promising phase 1 results paved the way for the ASCEMBL study, a phase 3 trial comparing asciminib to bosutinib in CML-CP patients who had experienced lack of efficacy or intolerance to ≥2 prior TKIs. Results from this study highlighted the superior efficacy of asciminib over bosutinib, with higher MMR rates and fewer adverse events leading to treatment discontinuation (11, 99).

Real-world experience with asciminib across various countries has further supported its efficacy in the third-line setting, with significant proportions of patients achieving MMRs, even among those with prior ponatinib exposure (100–104).

## Asciminib versus ponatinib, the better drug?

Ponatinib and high-dose asciminib demonstrate comparable efficacy in the context of the T315I mutation, as observed in the OPTIC and ASCEMBL trials, where each TKI was assessed as a third-line option. Ponatinib stands out as the preferred choice for patients with the F359 mutation (which is resistant to asciminib) and those with BCR::ABL >10% IS. Notably, in the OPTIC study, patients in the 45mg ponatinib group with >10% BCR::ABL achieved a higher MMR rate at 3 years compared to those on asciminib at 24 weeks. However, the longer follow-up duration with OPTIC warrants consideration (11, 84, 85). Conversely, Asciminib boasts a favorable vascular or cardiovascular safety profile and may be favored over ponatinib in patients with T315I mutation and significant vascular disease. This preference might evolve with accumulating long-term asciminib data. Asciminib could also be preferred in patients intolerant to previous 1G or 2G TKIs but who have achieved molecular response, as demonstrated in the ASCEMBL trial (105).

Compound mutations pose resistance to both TKIs. Given the absence of a direct head-to-head clinical trial comparing the efficacy and safety of ponatinib and asciminib, the optimal therapy decision should be individualized based on patient comorbidities and clinical judgment.

## Other therapeutic options

Omacetaxine mepesuccinate is a protein translation inhibitor that does not target the BCR-ABL kinase domain but induces apoptosis in BCR::ABL1 positive cells by downregulating MCL-1. It is effective against the T315I mutation and has been FDA-approved and available in the USA since 2012 for patients resistant or intolerant to ≥ 2 TKIs, including those with the T315I mutation (106, 107).

In a study involving 76 heavily pretreated evaluable patients with CML-CP, omacetaxine was administered as induction therapy at 1.25mg/m² BID subcutaneously for up to 14 consecutive days every 28 days until hematological response, followed by maintenance at 1.25mg/m² BID for up to 7 days every 28-day cycle. The study reported that 53 patients (70%) achieved a CHR, 14 patients (18%) achieved an MCyR, and 7 patients (9%) achieved a CCyR. Additionally, a partial cytogenetic response (PCyR) was achieved in 3.9% of patients, and a MCyR was achieved in 18.4%. Among 40 patients who had received 2 prior TKIs, 31 (78%) achieved a CHR, 10 (25%) an MCyR, and 5 (13%) a CCyR. Among 36 patients who had received 3 prior TKIs, 22 (61%) achieved a CHR, 4 (11%) an MCyR, and 2 (6%) a CCyR. For the 22 patients with T315I mutations at baseline, 18 (82%) achieved a CHR, 5 (23%) an MCyR, and 3 (14%) a CCyR. In 35 patients with CML-AP, 14.3% achieved a major hematologic response, and 11.3% achieved a CHR with no evidence of leukemia in 2.9% (106, 108).

While omacetaxine is generally well-tolerated and suitable for long-term administration, it can cause prolonged and severe myelosuppression. Hematological side effects occurring in ≥ 5% of patients included bone marrow failure (11%), thrombocytopenia (11%), and febrile neutropenia (7%). Common non-hematological side effects were diarrhea (43%), nausea (38%), fatigue (30%), infections (26%), pyrexia (22%), headache (22%), asthenia (22%), and arthralgia (20%). The median OS for evaluable patients and for those who received more than three cycles were 40.3 and 49.3 months, respectively. The median PFS for evaluable patients and for those who received more than three cycles of therapy were 9.6 and 9.9 months, respectively. Due to its moderate efficacy, with an overall PFS of less than 10 months and an OS of under 4 years, omacetaxine is reserved for patients who are unable to use any of the available TKIs and those who are not candidates for allogenic stem cell transplantation (108).

## Newer therapies

Despite the wide array of therapeutic options for CML-CP, some patients remain resistant or intolerant to all available TKIs, creating a need for the development of new TKIs, particularly for third-line therapy and patients with T315I mutations.

One promising candidate is olverembatinib (HQP1351), a third-generation orally active BCR-ABL1 TKI effective in CML regardless of genotype. It completed phase I and II trials involving 101 patients (86 with CML-CP and 15 with CML-AP). The median time from diagnosis to the initiation of olverembatinib therapy was 6 years. Among the patients, 63% had the T315I mutation, and 83% had received ≥2 lines of TKI therapy. After a median follow-up of 30 months, the CHR, CCyR, and MMR rates for CML-CP patients were 97%, 62%, and 51%, respectively. For those with the T315I mutation, these rates were 100%, 84%, and 72%, respectively. At three years, the PFS was 96.3% for CML-CP patients and 71.4% for those with CML-AP. Dosing was administered every other day for 28 days, with cohorts receiving 1–60mg. Thrombocytopenia of any grade and grade 3/4 was reported in 75.2% and 49.5% of patients, respectively. The most common non-hematological side effects were grade 1/2 skin hyperpigmentation and hypertriglyceridemia (109–111).

In 2023, the American Society of Hematology updated the results of the phase 2 study, confirming olverembatinib’s efficacy in TKI-resistant CML-CP, including T315I, compared to the best available therapy (imatinib, nilotinib, and dasatinib) (112). Encouraging results led to olverembatinib’s approval in China in November 2021 for treating adult patients with TKI-resistant CML-CP and T315I-mutated CML-AP, and again in November 2023 for treating adult patients with CML-CP resistant to and/or intolerant of first- and second-generation TKIs (113).

**Figure 1:**
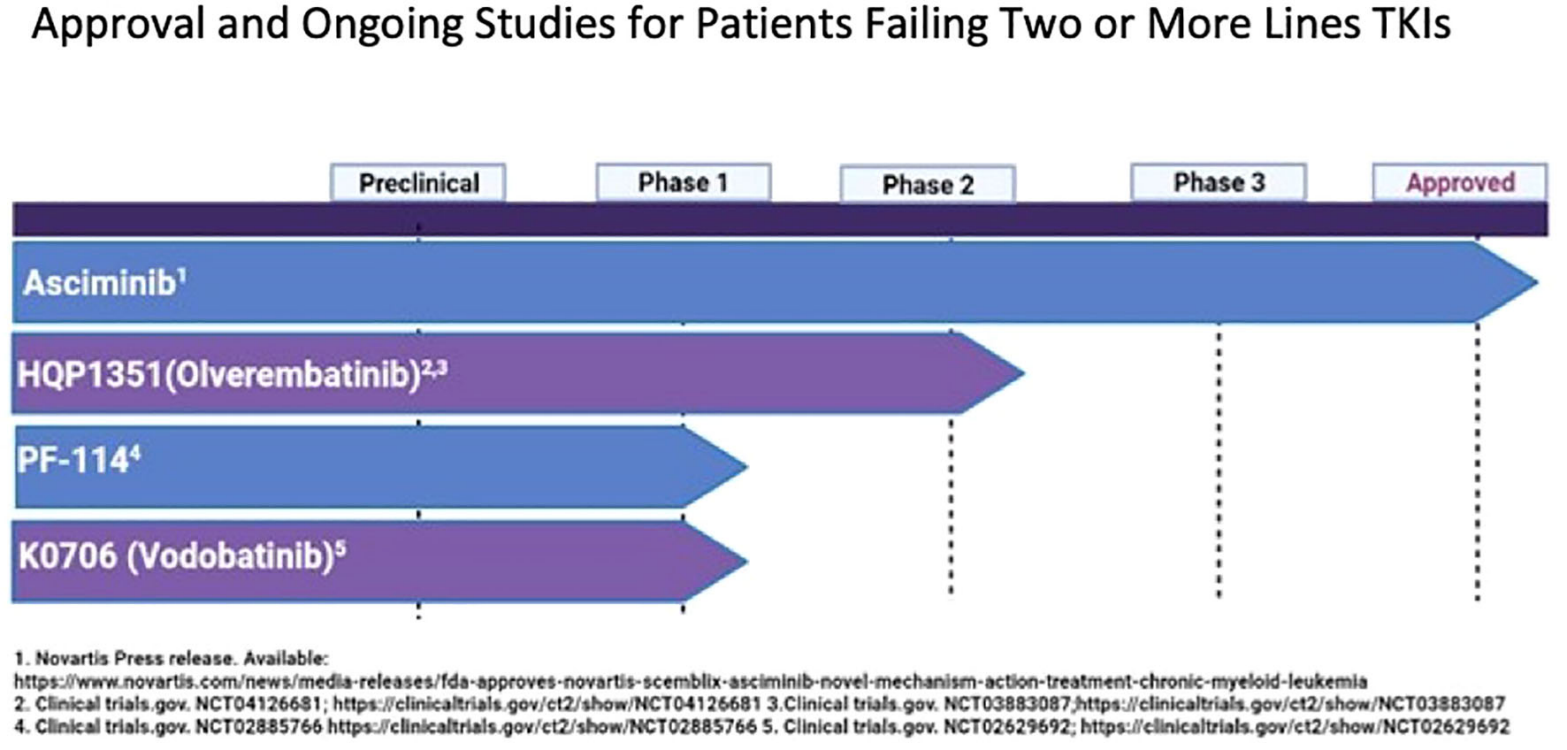
Approval and ongoing studies for patients failing 2 or more lines TKIs.

**Figure 2:**
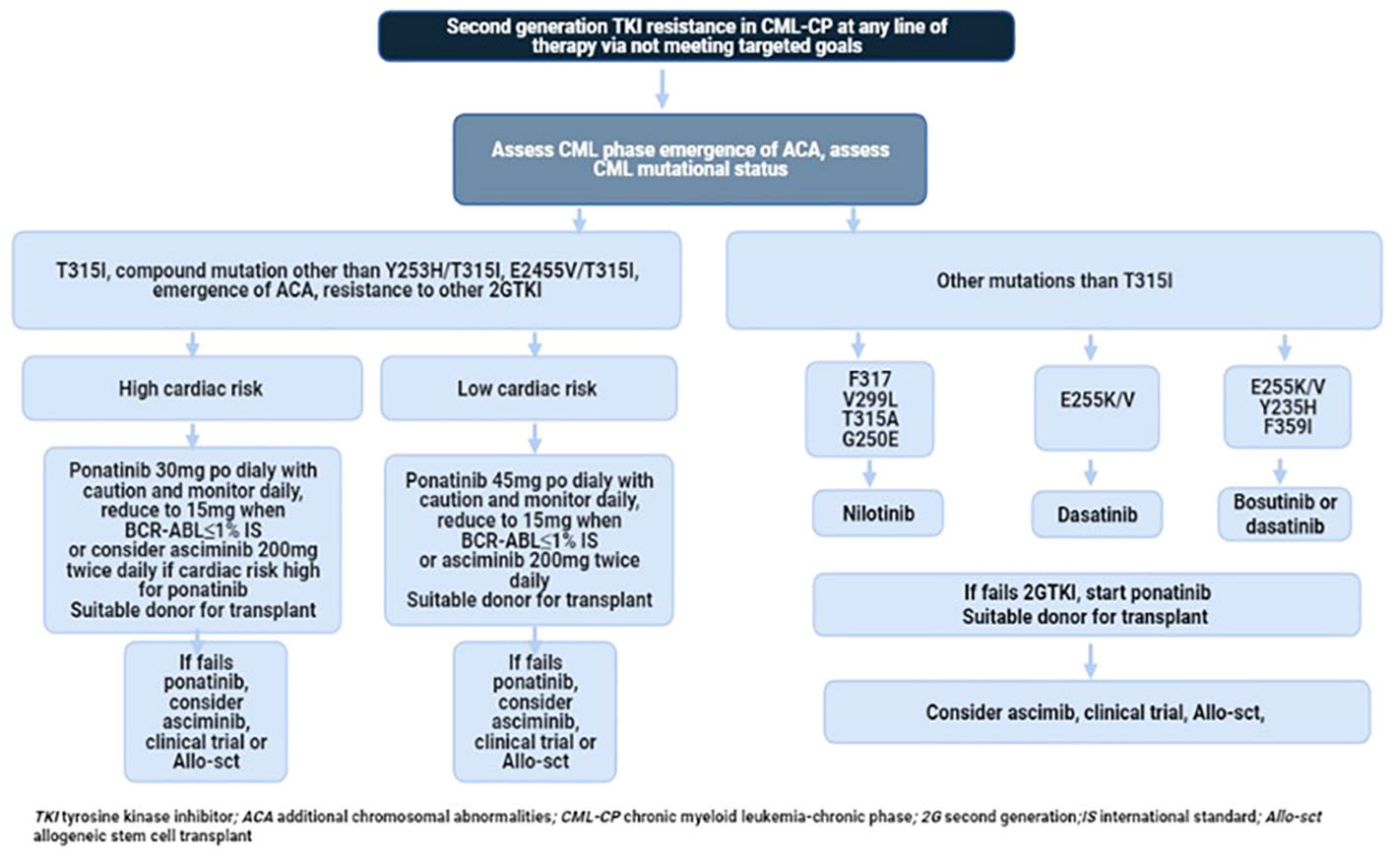
Recommended flow chart of management in patients with CP-CML who developed resistance to 2G TKI.

Current treatment options in this specific setting are suboptimal. Prospective clinical trials of single-agent TKIs in the third-line setting are detailed in **Table 3** and **Figure 1**. Additionally, several clinical trials are exploring combinations of TKIs with other agents targeting non-BCR-ABL-mediated CML leukemia stem cell (LSC) resistance. CML LSCs are not dependent on the kinase activity of BCR-ABL1 and are typically not eliminated by TKIs (117, 118). **Figure 2** provides a schematic algorithm for managing CML-CP patients who fail 2G TKI therapy.

**Table 3:** Ongoing clinical trials for BCR-ABL targeted therapies for CML in 3L+ context.

Drug	Trial/phase	Goal	Dose	Efficacy	Safety	Primary endpoints
HQP1351 (Olverembatinib) 3G BCR-ABL1 Inhibitor including T315I	NCT04126681 Phase II (109)	To evaluate the efficacy of HQP1351 in patients with CML-CP who are resistant and/or intolerant to 1G and 2GTKIs; mainly to use in 3L setting.	1–60 mg every other day	Median follow up 12.8month; 55 evaluable patients 94.5% achieved CHR; 81% achieved MCyR 60.5% achieved CCyR; 37.2% achieved MMR. Patients with T315I mutation had improved CHR, MCyR, CCyR and MMR	Any-grade and grade 3/4 thrombocytopenia reported in 75.2% and 49.5% of patients, respectively	EFS
	NCT03883087 Phase II (110)	To evaluate the efficacy of HQP1351 in patients with CML-CP and a T315I mutation	40 mg every other day for 28 days over 24 months	Median follow 7.9 month; 41 evaluable patients 96.8% achieved CHR; 75.6% achieved MCyR 65.9% and 9.85% achieved CCyR and PCyR, respectively 48.8% achieved MMR 3- and 6-month PFS was 100% and 96.7%	Frequent grade ≥ 3 treatment-related AEs were thrombocytopenia (48.8%), anemia (24.4%), neutropenia (19.5%), and leukopenia (12.2%). Frequent nonhematologic treatment-related all-grade AEs were skin pigmentation (53.7%) and elevated creatine kinase (48.8%), alanine aminotransferase (31.7%), and aspartate aminotransferase (26.8%)	MCyR
PF-114 BCR-ABL1 Inhibitor including T315I (114)	NCT02885766 Phase I/II (115)	To evaluate tolerability, safety, pharmacokinetics, and preliminary efficacy of PF-114 in patients with Ph+CML who are resistant to 2GTKIs or have the T315I mutation	50–750 mg daily	Follow-up of ≥ 6 months 6 of 11 patients achieved an MCyR and 4 patients achieved an MMR. 12 patients with T315I mutations, 3 achieved CHR and 4 patients achieved MCyR	Treatment-related AE grade 3 skin toxicity, mostly in the form of psoriasiform lesions, was reported in 11 patients receiving ≥ 400 mg	DLTs MTD
K0706 (Vodobatinib) BCR-ABL1 Inhibitor not including T315I (116)	NCT02629692 phase I/II	To determine safety, tolerability, pharmacokinetics, and activity in patients with CML or Ph+ALL CML Failure with ≥ 3 TKIs and/or patients with comorbidities that restrict the use of certain TKIs (dasatinib, nilotinib, and ponatinib)	Escalating dose of 12–240 mg daily	Mean duration of treatment 6.9 months; 12 of 27 evaluable patients. Eleven patients achieved and maintained a CCyR; Five achieved an MMR; and 2 achieved MR 4.5	Treatment-related events transient mild to moderate gastrointestinal disturbances (18.5%), General disorders [i.e.: myalgia, fatigue, asthenia] (15.7%), Neutropenia (12%) Thrombocytopenia (10%)	MTD TEAEs MCyR or partial cytogenetic response

CHR, complete hematologic response; MCyR, major cytogenetic response; CCyR, complete cytogenetic response; MMR, major molecular response; DLTs, dose limiting toxicity, MTD, maximum tolerated dose; TEAE, treatment-emergent adverse event.

Attempting TFR in patients who exhibit resistance to 2G TKIs is not currently recommended, although it remains a widely desired goal that is premature to undertake at this stage.

## When should an allogeneic transplant (allo-SCT) be considered?

Allo-SCT holds significant therapeutic implications as it represents a critical boundary between TKI treatment and transplantation. Before the era of TKIs like imatinib, allo-SCT was the only curative option and remains important today. Delaying transplant until all TKI options are exhausted is unfavorable, especially for patients with compound mutations or high risk of additional chromosomal aberrations (e.g., isochromosome 12, complex karyotype, trisomy 8, trisomy 19, monosomy 7, chromosome 3 abnormalities) (119).

Current ELN guidelines recommend considering allo-SCT for CML-CP patients resistant or intolerant to a second-line TKI or those with a T315I mutation (36, 37, 65). Further studies are needed to understand the implications of allo-SCT in the presence of somatic mutations such as ASXL1 or TP53 (22, 29).

The Center for International Blood and Marrow Research (CIBMTR) reported fewer than 300 transplants for CML-CP from 2014–2016. Compared to TKI therapy, allo-SCT achieves higher leukemia-free survival but is associated with nearly 20% transplant-related mortality at one year and decreased quality of life due to transplant complications like graft-versus-host disease. The five-year cumulative incidence of relapse (CIR) was 18%, with most relapses occurring in the first-year post-transplant, and the five-year overall survival (OS) was 68%. In 2020, CIBMTR reported fewer than 200 allotransplants for CML, mainly for accelerated and blast phases, while the European Bone Marrow Transplant (EBMT) registry reported nearly 400 transplants for CML, with almost half for CML-CP (120, 121). A recent Swedish study indicated three-year and five-year OS rates of approximately 85% and 96% for CML-CP, respectively, with a non-relapse mortality (NRM) of about 12% (122). Survival rates from various transplant registries are outlined in **Table 4**.

**Table 4:** Overall survival after allotransplant in CML-CP.

Registry/Study	Transplant Interval	N	Median age(yrs.)	Conditioning regimen	Donor	3-y OS (%)	5y OS (%)	10y OS (%)
CIBMTR (123)	1988–2003	3514	36	MA	REL	63	63	60
CIBMTR (123)	1988–2003	531	37	MA	UNR	58	55	50
Seattle (124)	1995–2000	131	43	MA	REL	86	NA	NA
German III (125)	1997–2004	148	41	MA	UNR	77	76	76
German registry (126)	1998–2004	1084	40	MA62%	REL61%	65	64	64
Japanese registry (127)	2000–2009	531	40	MA89%	UNR51%	85	85	78
CIBMTR (128)	2001–2010	224	24	MA	REL	85	83	NA
CIBMTR (128)	2001–2010	225	24	MA	UNR	72	68	NA
Korean (129)	2001–2012	47	32	MA77%	UNR43%	86	NA	NA
EBMT (130)	2002–2005	193	31	MA	REL	86	85	84
German study IV (131)	2003–2008	19	35	MA79%	REL53%	88%	NA	NA
German study IV (131)	2003–2008	37	38	MA65%	UNR70%	94%	NA	NA
Swedish (122)	2002–2017	56	40	MA44.6%	UNR 67.9%	–	96%	N/A

EBMT, European Group for Marrow and Blood Transplantation; CIBMTR, Center for International Blood and Marrow Transplantation; MA, myeloablative; REL, related donor; UNR, unrelated donor; NR, not reported; CML-CP, Chronic myeloid leukemia-chronic phase; NA, not available.

Advances in transplant techniques, including the use of matched related donors, preventing early relapses with donor lymphocyte infusion (DLI), stopping post-transplant immunosuppression, and treating with TKIs post-transplant, have improved three-year OS to above 85% and 15-year leukemia-free survival (LFS) to 80% (131–135). Although late relapses are rare, the risk of relapse continues indefinitely (135).

The impact of TKI use before and after transplant, as well as the number of TKIs used before transplant, on post-transplant OS remains unclear (134, 136). An EBMT score greater than 2 consistently shows an adverse impact on transplant outcomes and serves as a crucial tool for guiding transplant decisions (125, 133, 137). However, not all patients who fail TKI therapy are transplant candidates, especially older patients who may require reduced-intensity pre-transplant conditioning, which increases relapse risk (138). Therefore, the decision to proceed with a transplant in the chronic phase is complex, and early consideration for clinical trials should be optimized for patients not deemed transplant candidates.

## Conclusion

Despite advancements and a wide range of treatment options for CML-CP, 30–50% of patients experience failure with frontline imatinib within five years (123). With newer therapies in development primarily targeting the ATP-competitive BCR-ABL1, there should be a greater focus on strategically sequencing the available TKIs to optimize response and minimize the emergence of mutations and resistance. Asciminib has shown promising results and can address some of these unmet needs. Additionally, other pathways, including JAK/STAT, mTOR, and immune signaling, are promising potential targets for CML. Unfortunately, some patients may be unable to receive a second-generation TKI, ponatinib, or undergo allo-HSCT and may also be ineligible for clinical trials. In such cases, interferon alpha could be a viable option.

## Author contributions

BG: Conceptualization, Data curation, Formal analysis, Investigation, Project administration, Writing – original draft, Writing – review & editing. KC: Data curation, Validation, Writing – original draft, Writing – review & editing. AR: Data curation, Validation, Visualization, Writing – original draft, Writing – review & editing.
